# Supplementary material for: Transcriptome Analysis of the Adipose Tissue of Luchuan and Duroc Pigs
Source: Animals (Basel). 2022 Aug 31;12(17):2258. doi: 10.3390/ani12172258 (PMC9454924; doi:10.3390/ani12172258)
Supplement: Supplementary file 1 [file animals-12-02258-s001.zip › Supplementary File/Supplemental Table 1.pdf]

Table S1. Transcriptome data statistics.

Sample	Raw Reads	Clean Reads	Clean Reads Rate (%)	Q30	Mapped Reads	Mapping Rate
Duroc_1	47,456,140	45,889,484	96.7	95.91%	117,943,789	94.28%
Duroc_2	49,962,578	47,937,230	95.95	95.91%	118,682,360	94.68%
Duroc_3	46,846,584	45,082,868	96.23	95.98%	118,441,470	93.54%
Luchuan_1	45,890,832	43,859,842	95.57	95.90%	115,923,370	91.04%
Luchuan_2	48,171,536	46,262,262	96.04	95.88%	114,285,253	89.87%
Luchuan_3	47,453,720	45,651,590	96.2	95.88%	116,073,783	92.24%
